# Real-World Implementation of Molecular Testing and Biomarker-Guided Therapies in HR+/HER2− Metastatic Breast Cancer

**DOI:** 10.3390/cancers18213482

**Published:** 2026-10-28

**Authors:** Christine Deutschmann, Matthias Filipovic, Dagmar Baha, Leonhard Müllauer, Zsuzsanna Bago-Horvath, Daphne Gschwantler-Kaulich, Georg Pfeiler, Carmen Leser, Christian F. Singer

**Affiliations:** 1 Department of Obstetrics and Gynecology, Division of General Gynecology and Gynecologic Oncology, Medical University of Vienna, Waehringer Guertel 18-20, 1090 Vienna, Austria; 2 Department of Pathology, Medical University of Vienna, Waehringer Guertel 18-20, 1090 Vienna, Austria

**Keywords:** HR+/HER2− metastatic breast cancer, molecular testing, biomarker-guided therapy, PI3K/AKT pathway alteration, ESR1 mutation, BRCA1/2 mutation

## Abstract

**Background/Objectives:** The introduction of biomarker-guided therapies, including PI3K, AKT and PARP inhibitors as well as Selective Estrogen Receptor Degraders (SERDs), has expanded treatment options and delayed the need for chemotherapy in hormone receptor positive (HR+)/human epidermal growth factor receptor 2 negative (HER2-) metastatic breast cancer (mBC). Nevertheless, evidence regarding their implementation in routine clinical practice remains limited. This study evaluated the real-world use of molecular testing and biomarker-guided therapies in patients with HR+/HER2− mBC. **Methods:** Patients with HR+/HER2− mBC treated at the Medical University of Vienna, Austria, between July 2020 (EMA approval of alpelisib, marking the introduction of biomarker-guided therapies in HR+/HER2− mBC) and April 2025 were identified through a retrospective chart review. Patient and disease characteristics, molecular testing patterns, utilization of biomarker-guided therapies, and oncological outcome were assessed. Biomarker-guided therapies included alpelisib and capivasertib for PI3K/AKT pathway alterations, elacestrant for ESR1 mutations, and PARP inhibitors for BRCA1/2 mutations. Descriptive statistics summarized patient characteristics, molecular testing, and biomarker-guided therapies. Progression-free survival (PFS) and overall survival (OS) were estimated using the Kaplan–Meier method, and prognostic factors were evaluated using univariable and multivariable Cox regression analyses. **Results:** A total of 274 patients with HR+/HER2− mBC were included. 63.5% (174/274) of patients had undergone at least one molecular test. Overall, 59.8% (104/174) of patients harbored at least one actionable molecular alteration. Of these, 54.8% (57/104) received a biomarker-matched therapy. Of the 47 patients who had not received a matched therapy, 25 remained on first-line CDK4/6 inhibitor-based treatment and were therefore not yet candidates for subsequent biomarker-guided therapy. 12.8% (6/47) of patients with a targetable alteration did not receive a biomarker-matched therapy but transitioned to chemotherapy or an ADC. Concurrent actionable alterations were uncommon. Among 12 patients with concurrent PI3K/AKT pathway and ESR1 alterations, four received PI3K/AKT-directed therapy only and three ESR1-directed therapy only. Among four patients with concurrent PI3K/AKT pathway and BRCA1/2 alterations, three received PI3K/AKT-directed therapy only, whereas none received a PARP inhibitor. No patient with concurrent actionable alterations in different pathways received more than one biomarker-matched therapy sequentially. Biomarker-guided therapies achieved median treatment durations ranging from 3.5 to 14.5 months and postponed the need for chemotherapy or antibody-drug conjugates (ADCs) by a median of 9 months. **Conclusions:** Molecular testing was implemented in a substantial proportion of patients with HR+/HER2− mBC and identified actionable alterations in the majority of tested patients. The use of biomarker-guided therapies delayed treatment escalation to chemotherapy or ADCs.

## Simple Summary

New targeted therapies have expanded treatment options for patients with hormone receptor-positive, human epidermal growth factor receptor 2-negative metastatic breast cancer. Their use requires molecular testing to identify eligible patients, while the growing number of treatment options has increased the complexity of treatment sequencing. Given these challenges, understanding how molecular testing and biomarker-guided therapies have been adopted in routine clinical practice is important. We evaluated their real-world use at an academic center. Molecular testing was broadly implemented. More than half of patients with actionable alterations received matched therapies, but some patients died or were lost to follow-up before treatment was initiated, emphasizing the importance of early use of biomarker-guided therapies. Our findings also highlight challenges in treatment sequencing, as no patient with multiple actionable alterations received sequential biomarker-guided therapies targeting more than one alteration. These findings provide real-world evidence that may help optimize the delivery of biomarker-guided therapies for patients with metastatic breast cancer.

## 1. Introduction

The introduction of biomarker-guided therapies for hormone receptor-positive (HR+)/human epidermal growth factor receptor 2 negative (HER2−) metastatic breast cancer (mBC), including phosphoinositide 3-kinase (PI3K) [1,2], protein kinase B (AKT) [3] and poly(ADP-ribose) polymerase (PARP) inhibitors [4,5] as well as selective estrogen receptor degraders (SERDs) [6], has prolonged progression-free survival (PFS), expanded treatment options, and delayed the need for chemotherapy [7]. However, the increasing number of biomarker-guided therapies has also introduced new clinical challenges.

Molecular testing has become an integral component of treatment selection in HR+/HER2− mBC [8,9,10,11]. Actionable genomic alterations are commonly identified using targeted polymerase chain reaction (PCR)-based assays or next-generation sequencing (NGS). PCR is a rapid, highly sensitive, and cost-effective method for detecting predefined hotspot mutations. In contrast, NGS enables comprehensive profiling of multiple clinically relevant genes but is associated with greater technical complexity, higher costs, and longer turnaround times [12]. Furthermore, interpretation of NGS results requires specialized molecular pathology expertise to classify genomic alterations according to their clinical actionability, which may not be readily available in all treatment centers [13].

The optimal specimen source and timing of molecular testing remain important clinical questions. Tissue-based molecular profiling is well established. However, circulating tumor DNA (ctDNA) provides a minimally invasive alternative that captures spatial and temporal tumor heterogeneity by detecting DNA released from multiple tumor clones throughout disease evolution [14]. Consequently, ctDNA has become the preferred testing modality for several actionable alterations, particularly ESR1 [15], whereas tissue-based testing may remain preferable for PTEN because of its higher analytical sensitivity [16].

Furthermore, co-occurring actionable alterations are increasingly recognized in HR+/HER2− mBC. Large genomic studies have shown that approximately one-third of patients with PI3K/AKT pathway alterations also harbor concurrent ESR1 mutations [17] and the prevalence of these co-alterations increases with successive endocrine treatment lines [18]. However, prospective evidence guiding the optimal management and sequencing of targeted therapies in patients with multiple actionable alterations remains limited [19].

Collectively, these challenges may hinder the integration of molecular testing and biomarker-guided therapies into routine clinical practice. Real-world data on their implementation remain scarce. To address this gap, we evaluated the real-world use of molecular testing and biomarker-guided therapies in patients with HR+/HER2− mBC.

## 2. Methods

Patients with HR+/HER2− mBC treated at the Department of Obstetrics and Gynecology, Medical University of Vienna, Austria, between 30 July 2020 (EMA approval of alpelisib, marking the introduction of biomarker-guided therapies in HR+/HER2− mBC), and 9 April 2025, were identified through a retrospective chart review. Patients with a second primary malignancy were excluded.

HR+/HER2− status was determined according to contemporary ASCO/CAP guidelines [20,21] based on pathology reports available in the medical records. Patient and disease characteristics, molecular testing patterns, the use of biomarker-guided therapies, and oncological outcomes were assessed. Patients were followed until death or the database cutoff date of 9 April 2025.

Molecular analyses were performed as part of routine clinical care using different commercially available assays, including the Oncomine Comprehensive Assay v3 and Oncomine Focus Assay (Thermo Fisher Scientific), TruSight Oncology 500 (Illumina), AmoyDx Comprehensive Panel (Amoy Diagnostics), Hedera Profiling 3 ctDNA test panel (Hedera Dx), and Hereditary Cancer Solution (SOPHiA Genetics) as well as targeted PCR followed by Sanger sequencing (see Table S1). Patients could undergo repeated molecular testing at different time points, using different specimens and testing platforms. Therefore, specimen type and testing methodology were evaluated at the level of each molecular analysis. As the molecular targets covered varied across assays, not all patients were evaluated for every actionable alteration. Consequently, biomarker-specific prevalence was calculated using only patients with an evaluable result for the respective alteration as the denominator. Alterations for which approved biomarker-guided treatment options were available during the study period were considered actionable and included alterations in PIK3CA, AKT1, PTEN, ESR1, and BRCA1/2.

Biomarker-guided therapies included alpelisib and capivasertib in combination with endocrine therapy for PI3K/AKT pathway alterations, elacestrant for ESR1 mutations, and PARP inhibitors for BRCA1/2 mutations.

Endocrine sensitivity and resistance were classified according to the latest ABC guidelines [22].

PFS was defined as the time from initiation of the respective biomarker-guided therapy to disease progression, death from any cause, or last follow-up, whichever occurred first. Overall survival (OS) was defined as the time from initiation of the respective biomarker-guided therapy to death from any cause or last follow-up.

Survival analyses were based on the first biomarker-guided therapy received.

Continuous variables are presented as median and interquartile range (IQR), whereas categorical variables are reported as absolute and relative frequencies. PFS and OS were estimated using the Kaplan–Meier method. Associations between clinical and molecular variables and PFS and OS were evaluated using univariable Cox proportional hazards regression models, followed by multivariable Cox regression analyses to identify independent prognostic factors. Candidate variables, including treatment line, concurrent actionable alterations, variant allele frequency (VAF), number of involved organ systems, and endocrine sensitivity, were selected a priori based on clinical relevance. The proportional hazards assumption was assessed using Schoenfeld residuals. Missing data were not imputed, and analyses were performed using available cases. Given the exploratory nature of the analyses, no adjustment for multiple comparisons was performed. *p*-values < 0.05 were considered statistically significant. Statistical analyses were performed using SPSS version 31.0.

## 3. Results

### 3.1. Patient and Disease Characteristics

A total of 274 patients with HR+/HER2− mBC were included in the analysis. The median age was 67 years (Q1–Q3: 56, 80), and all patients were female. Most patients presented with recurrent metastatic disease (66.1%). Endocrine-sensitive disease was observed in 42.3% (116/274) of patients, while 39.8% (109/274) and 17.9% (49/274) had secondary and primary endocrine resistance, respectively. During follow-up, 68 patients died (see Table 1).

### 3.2. Molecular Testing, Genomic Alterations, and Mutation Patterns

After a median follow-up of 27.9 months (Q1–Q3: 9.0–47.0) from diagnosis of metastatic disease, 63.5% (174/274) of patients had undergone at least one molecular test. Among patients who underwent molecular testing, 75.9% (132/174) underwent a single mutation analysis, whereas 18.4% (32/174), 3.4% (6/174), 1.7% (3/174), and 0.6% (1/174) underwent two, three, four, and six analyses, respectively, reflecting repeated testing at different time points and/or from different specimens.

Overall, 36.5% (100/274) of patients did not undergo molecular testing. Patient and disease characteristics were similar between patients who did and did not undergo molecular testing, except for older age (median, 67 vs. 59 years; *p* < 0.001) and a higher proportion of patients treated in the first-line setting (81.0% vs. 33.9%; *p* < 0.001) among those who did not undergo testing (see Table S2). Notably, all patients without molecular testing had initiated their ongoing treatment before the EMA approval of the PIK3CA inhibitor inavolisib. Consequently, no clinical indication for molecular testing prior to first-line treatment of metastatic disease existed at the time of treatment initiation in these patients.

The median time from diagnosis of metastatic disease to first molecular testing was 2 months (Q1–Q3: 0–19).

A total of 232 molecular analyses were evaluated, of which 74.2% (170/229) were tissue-based and 76.1% (172/226) were performed using NGS.

Overall, 59.8% (104/174) of patients harbored at least one actionable molecular alteration (see Table 2).

Alterations in the PI3K/AKT pathway were the most common, including PIK3CA mutations in 42.3% (69/163), PTEN alterations in 11.7% (9/77), and AKT1 alterations in 4.0% (5/125) of patients. Among the 79 patients with a PI3K/AKT pathway alteration, 66 harbored an isolated PIK3CA mutation, 6 an isolated PTEN alteration, and 3 an isolated AKT1 alteration. Co-occurring alterations in the PI3K/AKT pathway were identified in 2 patients with concurrent PIK3CA and PTEN alterations, 1 patient with concurrent PIK3CA and AKT1 alterations, and 1 patient with concurrent AKT1 and PTEN alterations.

ESR1 mutations were detected in 22.0% (27/123) and BRCA1/2 mutations in 18.9% (14/74) of patients.

**Figure 1:**
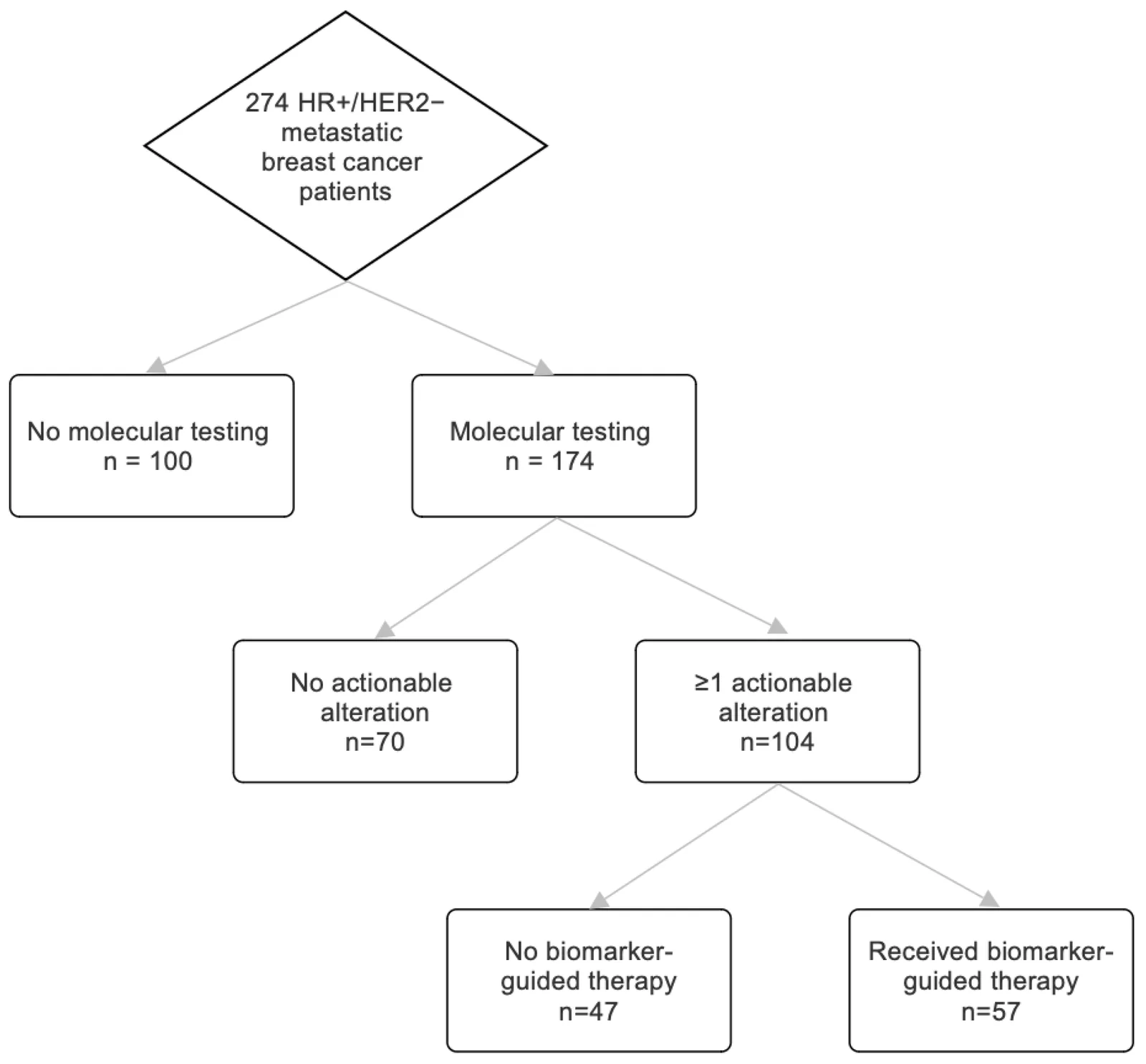
Study schema of molecular testing and biomarker-guided therapies in HR+/HER2− metastatic breast cancer. The categories represent sequential steps in the testing and treatment pathway rather than predefined comparative patient groups. HR hormone receptor, HER2 Human Epidermal growth factor Receptor 2.

**Figure 2:**
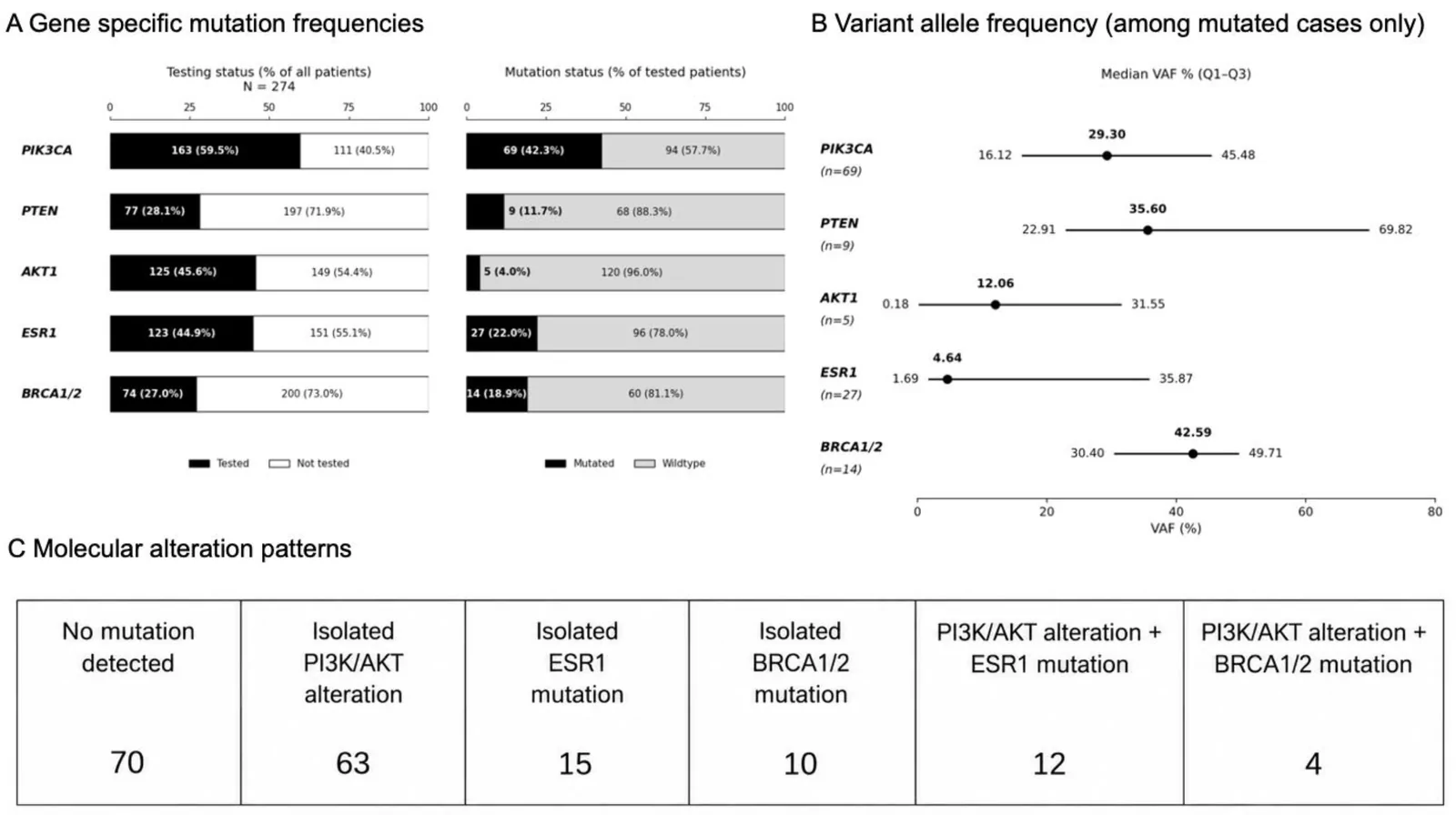
Molecular testing uptake and results, (**A**) Testing rates and mutation frequencies for PIK3CA, PTEN, AKT1, ESR1, and BRCA1/2. (**B**) Distribution of variant allele frequencies among patients with detected mutations. (**C**) Distribution of isolated and co-occurring molecular alterations among tested patients. PI3K/AKT pathway alterations include mutations in PIK3CA, PTEN and AKT1. VAF variant allele frequency.

Concurrent actionable alterations in different pathways were uncommon: 12 patients harbored both PI3K/AKT pathway and ESR1 alterations, while 4 patients had concurrent PI3K/AKT pathway and BRCA1/2 alterations. No patients harbored concurrent ESR1 and BRCA1/2 alterations or simultaneous alterations in the PI3K/AKT pathway, ESR1, and BRCA1/2 (see Figure 1 and Figure 2, Table 2 and Table S3).

### 3.3. Uptake of Biomarker-Matched Therapies Following Molecular Profiling

54.8% (57/104) of patients with a targetable alteration received a biomarker-matched therapy.

Among patients harboring a PI3K/AKT pathway alteration, 41.8% (33/79) received a PI3K/AKT-directed therapy, including 27 patients treated with alpelisib plus endocrine therapy and 3 patients treated with capivasertib plus endocrine therapy and 3 patients who received alpelisib and capivasertib plus endocrine therapy sequentially.

ESR1-directed therapy with elacestrant was administered to 59.3% (16/27) of patients with an ESR1 mutation, while 57.1% (8/14) of patients with a BRCA1/2 mutation received a PARP inhibitor.

Among the 12 patients with concurrent PI3K/AKT pathway and ESR1 alterations, four received PI3K/AKT-directed therapy only and three received ESR1-directed therapy only. Among the four patients with concurrent PI3K/AKT pathway and BRCA1/2 alterations, three received PI3K/AKT-directed therapy only, whereas none received a PARP inhibitor. No patient with concurrent actionable alterations in different pathways received two or more biomarker-matched therapies sequentially.

**Figure 3:**
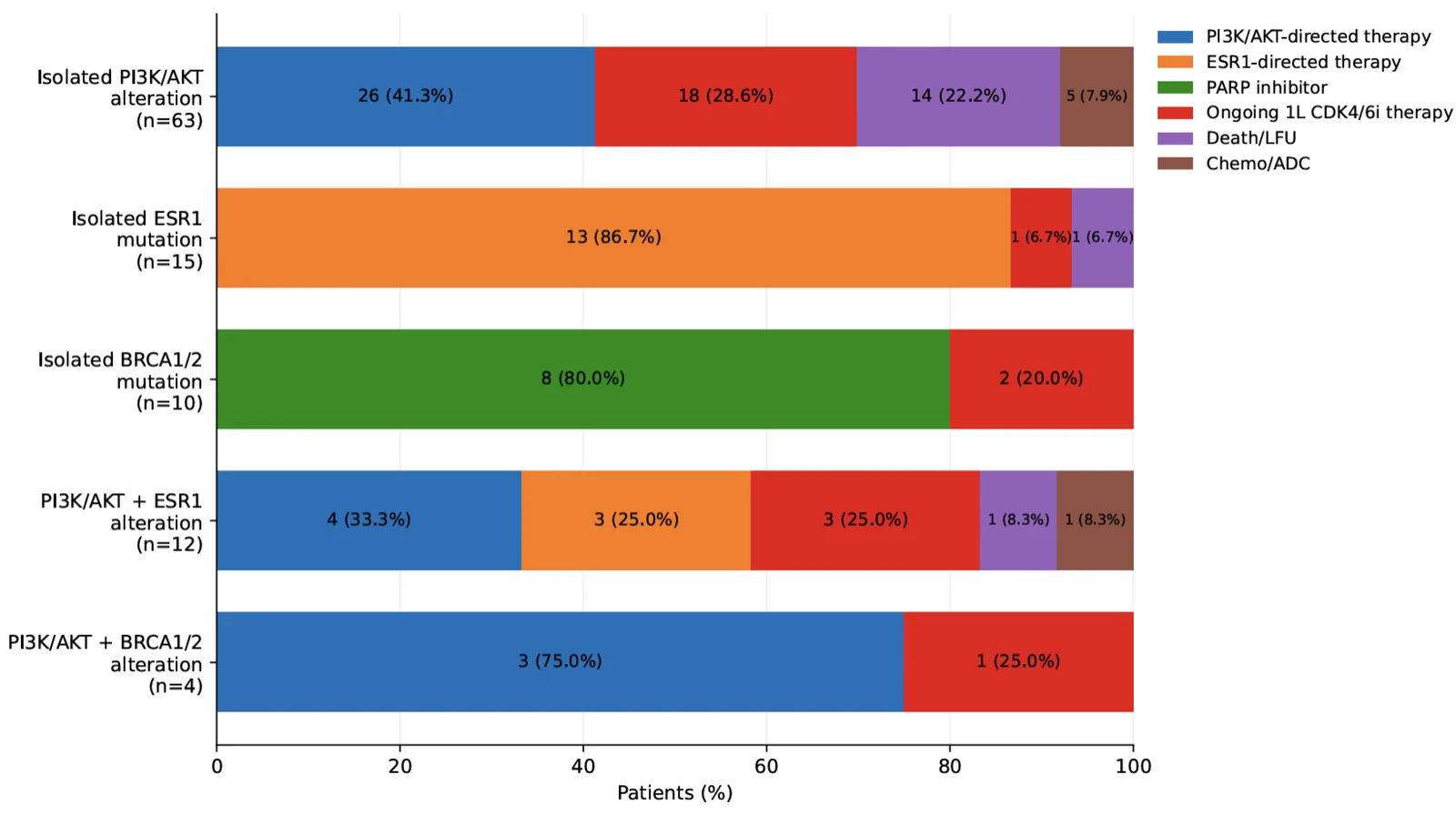
Uptake of biomarker-guided therapies according to molecular alteration pattern, 1L first line, LFU lost to follow-up, ADC antibody drug conjugate.

Overall, 45.2% (47/104) of patients harboring an actionable alteration had not received a biomarker-guided therapy by the end of follow-up. However, 53.2% (25/47) remained on first-line CDK4/6 inhibitor-based treatment and were therefore not yet candidates for subsequent biomarker-guided therapy. Among the remaining 22 patients, 16 (72.7%) died or were lost to follow-up before initiation of matched therapy, while 6 (27.3%) transitioned to chemotherapy or an ADC (see Figure 3). The specific reasons for transitioning to chemotherapy or an ADC could not be determined retrospectively.

Targeted therapy deferred the initiation of ADCs or chemotherapy by a median of 9 months (Q1–Q3: 6–17 months).

### 3.4. Survival Outcomes and Prognostic Factors During Biomarker-Guided Therapy

Survival analyses were based on the first biomarker-guided therapy received.

Median PFS was 10.9 months (95% CI, 7.5–14.0) for alpelisib plus endocrine therapy, 5.2 months (95% CI, 3.4–not reached) for capivasertib plus endocrine therapy, 3.5 months (95% CI, 0.6–5.1) for elacestrant, and 14.5 months (95% CI, 3.4–not reached) for PARP inhibitors. Corresponding median OS values were 47.8 months (95% CI, 12.3–not reached), not reached, 9.7 months (95% CI, 0.6–not reached), and 34.3 months (95% CI, 3.4–not reached), respectively.

**Figure 4:**
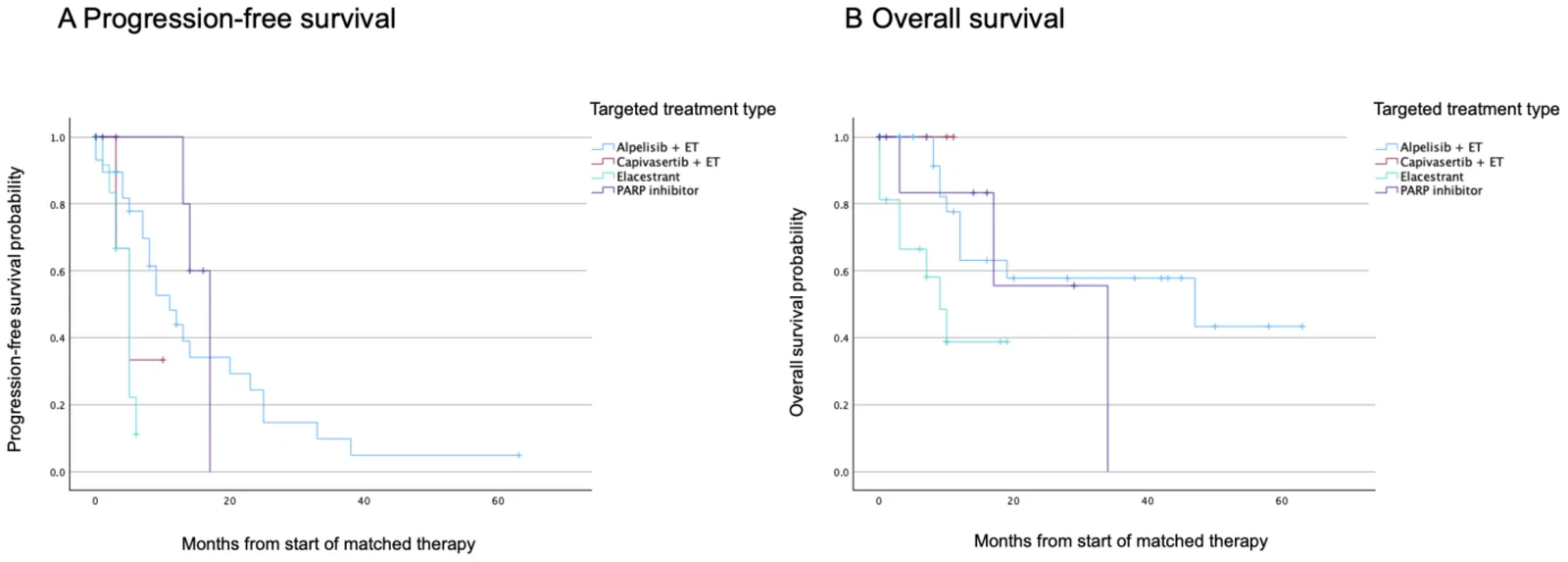
Kaplan–Meier estimates of (**A**) progression-free and (**B**) overall survival. Survival analyses were based on the first biomarker-guided therapy received, ET endocrine treatment.

Treatment line, concurrent actionable alterations, VAF, number of involved organ systems, and endocrine sensitivity were evaluated as potential predictors of PFS and OS using univariable and multivariable Cox proportional hazards regression models. None of the investigated variables were significantly associated with PFS. For OS, treatment line (HR 1.77, 95% CI 1.13–2.79; *p* = 0.013) and VAF (HR 0.68 per 10 percentage point increase, 95% CI 0.49–0.94; *p* = 0.021) were significantly associated with outcome in univariable analyses, with later treatment lines being associated with an increased risk of death and higher VAF being associated with a reduced risk of death. Concurrent actionable alterations, number of involved organ systems, and endocrine sensitivity were not associated with OS. In multivariable analysis, no variable remained independently associated with OS (see Figure 4, Table S4 and Figure S1). The proportional hazards assumption was generally met; however, a potential violation was observed for concurrent actionable alterations in the PFS analysis. The corresponding estimate was retained for exploratory purposes and should therefore be interpreted with caution.

## 4. Discussion

Current ESMO guidelines for the treatment of HR+/HER2− mBC recommend assessment of PIK3CA, AKT1, PTEN, ESR1, BRCA1/2, and PALB2 alterations to identify patients eligible for biomarker-guided therapies [23]. However, translating these recommendations into routine clinical practice is challenging because it requires timely molecular testing, accurate interpretation of increasingly complex molecular profiles, and integration of molecular findings into treatment selection and sequencing [7]. Given these challenges, understanding how molecular testing and biomarker-guided therapies have been adopted in routine clinical practice is increasingly important. Yet, real-world evidence describing current practice remains scarce [24]. This study evaluated the real-world use of molecular testing and biomarker-guided therapies in patients with HR+/HER2− mBC.

Overall, molecular testing was broadly implemented and performed in 63.5% of patients. Actionable molecular alterations were identified in 59.8% of tested patients. Patients who did not undergo molecular testing were significantly more likely to remain in the first-line setting (81.0% vs. 33.9%), and all had initiated their ongoing first-line treatment before the EMA approval of the PI3K inhibitor inavolisib, when molecular profiling prior to first-line treatment was not yet clinically indicated. Thus, the observed lack of molecular testing in this study primarily reflects historical treatment patterns rather than incomplete implementation of molecular testing. Untested patients were also significantly older than tested patients. Although the reason for this difference remains unclear and potential age-related selection in molecular testing cannot be excluded, older patients, who may have a greater comorbidity burden, may particularly benefit from biomarker-guided treatment options that can offer a more favorable toxicity profile, allow oral administration, and postpone the need for chemotherapy. Thus, older age should not represent a barrier to molecular testing.

Most molecular analyses were tissue-based and performed NGS. The high utilization of NGS reflects the increasing need for comprehensive molecular profiling as the number of clinically actionable biomarkers continues to expand. However, the optimal testing source depends on the molecular alteration. PIK3CA and AKT1 alterations arise early and remain stable throughout disease progression, resulting in high concordance between tissue- and plasma-based testing [25,26,27]. In contrast, tissue-based testing appears to have greater sensitivity for detecting PTEN alterations [16]. ESR1 mutations arise as a mechanism of acquired resistance during aromatase inhibitor therapy and increase with successive endocrine treatment lines [28]. Consequently, ctDNA has become the preferred testing modality for ESR1, and current ASCO guidelines recommend repeated testing at disease progression in patients who initially remain ESR1 wild-type [15]. Consistent with these recommendations, almost one quarter of patients in our cohort underwent repeated molecular analyses.

PIK3CA mutations represented the most common actionable molecular alterations in our cohort (42.3%), followed by ESR1 (22.0%) and BRCA1/2 mutations (18.9%). These prevalence rates were consistent with those reported in pivotal clinical trials and contemporary real-world studies, supporting the representativeness of our cohort [1,2,4,5,6,18].

Although concurrent actionable alterations were uncommon, the most frequent combination involved the PI3K/AKT pathway and ESR1, whereas concurrent ESR1 and BRCA1/2 alterations or simultaneous alterations involving the PI3K/AKT pathway, ESR1, and BRCA1/2 were not observed. This pattern is consistent with recent genomic studies showing that approximately one-third of patients with PI3K/AKT pathway alterations also harbor ESR1 mutations, whereas concurrent BRCA1/2 alterations remain uncommon [17]. Similarly, a tissue-based genomic analysis demonstrated increasing rates of concurrent ESR1 and PI3K/AKT pathway alterations with successive treatment lines [18] likely reflecting the acquisition of ESR1 mutations during endocrine therapy. These findings support comprehensive molecular profiling at metastatic diagnosis followed by repeating ESR1 testing during disease progression, in line with current ASCO recommendations [15].

Biomarker-guided therapies were implemented in more than half of patients harboring actionable molecular alterations. Uptake was comparable across biomarker groups, with 41.8% of patients harboring PI3K/AKT pathway alterations receiving PI3K/AKT-directed therapy, 59.3% of patients with ESR1 mutations receiving elacestrant, and 57.1% of patients with BRCA1/2 mutations receiving PARP inhibitor therapy. Moreover, our findings highlight the increasing complexity of treatment selection in patients with concurrent actionable alterations. In our cohort, no patient with alterations involving different molecular pathways received sequential biomarker-guided therapies targeting more than one alteration. While the specific reasons for treatment choices could not be determined retrospectively, potential explanations include limited clinical evidence regarding the optimal sequencing of targeted therapies, transition to chemotherapy or ADCs in patients considered endocrine-insensitive, reimbursement considerations, physician decision-making, and patient attrition [24]. Importantly, this study reflects an early period in the implementation of several biomarker-guided therapies, and some patients remained on an ongoing targeted treatment at the end of follow-up without an indication for treatment change. Notably, available evidence suggests that co-occurring alterations do not necessarily compromise treatment efficacy. Retrospective analyses demonstrated that alpelisib plus fulvestrant retains activity in tumors harboring concurrent PIK3CA and ESR1 mutations, while post hoc analyses of the phase III EMERALD trial showed that the clinical benefit of elacestrant was maintained in PIK3CA-mutated tumors [19]. Comparable evidence for PARP inhibitors is currently lacking. Prospective studies are therefore needed to define the optimal treatment sequence in patients with multiple actionable alterations.

The median PFS with alpelisib plus endocrine therapy, capivasertib plus endocrine therapy, elacestrant, and PARP inhibitors was 10.9, 5.2, 3.5, and 14.5 months, respectively, while the corresponding median OS was 47.8 months, not reached, 9.7 months, and 34.3 months. Overall, these outcomes were broadly consistent with those reported in the pivotal SOLAR-1 [1], CAPItello-291 [3], EMERALD [6] and OlympiAD [4] trials, although the survival estimates were descriptive and should not be interpreted as direct comparisons of treatment efficacy given the small and unequal treatment groups in this retrospective real-world cohort. Among the investigated clinicopathological and molecular variables, only treatment line and VAF were associated with OS in univariable analyses. The association between later treatment lines and inferior survival is consistent with the progressive development of endocrine resistance and the declining efficacy of successive endocrine-based therapies in HR+/HER2− mBC [29]. Unexpectedly, higher baseline VAF was associated with improved OS. Although the presence of actionable genomic alterations is an established predictive biomarker for targeted therapies, the prognostic significance of baseline VAF remains poorly defined. Biologically, VAF may reflect the clonality of a targetable genomic alteration [30]. However, clinically meaningful baseline VAF thresholds have not yet been established in breast cancer. Previous studies have primarily investigated longitudinal ctDNA monitoring, showing that dynamic suppression of ESR1 or PIK3CA mutations during treatment is associated with improved clinical outcome [31,32]. Given this limited evidence, together with the small sample size and number of events in our study, particularly within individual treatment subgroups, the observed association should be considered exploratory and hypothesis-generating and requires external validation in larger cohorts.

Beyond prolonging disease control, postponing the need for chemotherapy represents an important clinical objective of biomarker-guided therapies. In our cohort, targeted treatment delayed the need for chemotherapy or ADCs by a median of 9 months. Although time to chemotherapy has not been established as a standard efficacy endpoint and has only been reported in selected pivotal trials [1,3,6], a similar delay has been observed in studies evaluating alpelisib, capivasertib, and elacestrant. Moreover, the consistent prolongation of disease control across pivotal trials [1,2,3,4,5,6] together with recent real-world evidence demonstrating that most patients receive multiple lines of endocrine-based therapy before chemotherapy [33] supports the concept that biomarker-guided therapies can postpone the need for chemotherapy.

Importantly, the observed uptake of biomarker-guided therapy should be interpreted in the context of treatment eligibility. More than half of patients with actionable alterations who had not received matched therapy remained on effective first-line CDK4/6 inhibitor-based treatment and were therefore not yet candidates for subsequent biomarker-guided therapy. Of the remaining patients, a substantial proportion had died or was lost to follow-up before treatment initiation, reflecting the well-recognized attrition of patients across successive treatment lines and emphasizing the importance of implementing targeted therapies early in the treatment course [34].

Furthermore, some patients with actionable alterations transitioned directly to chemotherapy or ADCs without receiving biomarker-guided therapy. While the specific reasons for subsequent treatment selection could not be determined retrospectively, these findings may highlight the ongoing challenge of defining endocrine insensitivity in clinical practice and determining the optimal timing of transitioning from endocrine-based treatment strategies to chemotherapy or ADCs [22]. This uncertainty is additionally reflected in recent real-world data from the United States, where 86.4% of patients received two or more lines of endocrine-based therapy before initiating chemotherapy, whereas 13.6% started chemotherapy after only one line of CDK4/6 inhibitor plus endocrine therapy [33]. Notably, approximately one quarter of patients were treated with endocrine therapy with or without targeted therapy after having received chemotherapy. Identifying patients who require early chemotherapy to achieve rapid disease control but may subsequently benefit from endocrine-based rechallenge warrants further investigation.

Beyond currently established actionable biomarkers, emerging preclinical evidence suggests that additional molecular targets may further expand treatment options in breast cancer. For example, inhibition of the DNA repair factor CSA/ERCC8 has been shown to impair breast cancer cell proliferation and enhance sensitivity to cytotoxic agents [35].

This study has several limitations. Its retrospective single-center design introduces the potential for selection bias, as also reflected by differences in age and treatment line between patients who did and did not undergo molecular testing. The relatively small biomarker- and treatment-defined subgroups and limited number of events reduced statistical power, particularly for multivariable survival analyses. In addition, the potential violation of the proportional hazards assumption for concurrent actionable alterations limits the interpretation of the respective PFS estimate. Molecular testing was performed using different platforms in routine clinical practice, and only genes included in the respective assay could be analyzed. In addition, PALB2 alterations were not routinely assessed during the study period despite current guideline recommendations, and germline BRCA1/2 mutations may have been underreported because some patients requested restricted access to their genetic test results within the electronic medical record. Finally, comparisons with pivotal clinical trials should be interpreted cautiously because of differences in study design, patient selection, and evolving treatment standards.

## 5. Conclusions

In this real-world cohort of patients with HR+/HER2− mBC, molecular testing was implemented in a substantial proportion of patients and identified actionable alterations in the majority of tested cases. Biomarker-guided therapies were adopted in more than half of patients harboring actionable alterations and delayed the need for chemotherapy or ADCs. A substantial proportion of patients with actionable alterations died or were lost to follow-up before receiving biomarker-guided therapy, emphasizing the importance of implementing targeted therapies early in the treatment course. Moreover, no patient with concurrent actionable alterations received sequential biomarker-guided therapies targeting more than one molecular alteration, and some patients transitioned directly to chemotherapy or ADCs. Together, these findings highlight the complexity of treatment sequencing and defining endocrine insensitivity. Addressing these unresolved questions will be essential to further optimize the implementation of targeted treatments in HR+/HER2− mBC.

## Figures and Tables

**Table 1:** Patient and disease characteristics.

Patient and Disease Characteristics	Patients, *n* = 274 (%)
**Age,** years, median (Q1–Q3)	67 (56, 80)
**Female sex**	274 (100%)
**Timing of metastatic disease**	
- De novo	93 (33.9%)
- Recurrent	181 (66.1%)
**Endocrine sensitivity**	
- Endocrine sensitive disease	116 (42.3%)
- Primary endocrine resistance	49 (17.9%)
- Secondary endocrine resistance	109 (39.8%)
**Death during follow-up**	68 (24.8%)

**Table 2:** Molecular testing, genomic alterations, and alteration patterns.

Parameter	Patients, *n* = 274 (%)
**Molecular testing**	
- Total number of mutation analyses in the dataset	232
- Patients with ≥1 mutation analyses	174 (63.5%)
- Patients without mutation analyses	100 (36.5%)
- Number of mutation analyses per patient, median (Q1–Q3)	1 (1, 1)
- Time from mBC diagnosis to first mutation analysis, months, median (Q1–Q3) *	2 (0–19)
**Specimen type**	
- Tissue	170 (74.2%)
- Blood	59 (25.8%)
- Missing	3
**Genomic testing methodology**	
- PCR	54 (23.9%)
- NGS	172 (76.1%)
- Missing	6

* Only considering patients with ≥1 mutation analyses, Specimen type and testing methodology are reported per molecular analysis. Q1-Q3 First to third quartile, PCR Polymerase Chain Reaction, NGS Next-generation sequencing.

## Data Availability

Data are not available for privacy reason.

## Supplementary Materials

The following supporting information can be downloaded at: https://www.mdpi.com/article/10.3390/cancers18213482/s1, Figure S1: Forest plots of univariable and multivariable Cox regression models for progression-free and overall survival. Multivariable analyses included 34 patients with complete data for all covariates, with 26 PFS events and 15 OS events. PFS progression free survival, OS overall survival, VAF variant allele frequency; Table S1: Overview of molecular assays used for biomarker assessment, SNV single-nucleotide variant, in-del insertion/deletion, CNV copy-number variation, TMB tumor mutational burden, MSI mi-crosatellite instability, cfDNA cell-free DNA, SNP single-nucleotide polymorphism; Table S2: Characteristics of patients with and without mutation analysis, * including patients with >1st treatment line, ET endocrine treatment, ADC antibody drug conjugate; Table S3: Mutation analyses, continued from Table 2. Treatment line and specimen type are reported per molecular analysis. Table S4: Utilization and treatment characteristics of biomarker-guided therapies. Survival analyses were performed according to the first biomarker-guided therapy received, ET endocrine treatment.

## Abbreviations

The following abbreviations are used in this manuscript:

ADC	Antibody drug conjugate
AKT1	Protein kinase B 1
ASCO	American Society of Clinical Oncology
CAP	College of American Pathologists
CDK4/6	Cyclin-dependent kinases 4 and 6
Chemo	Chemotherapy
CI	Confidence interval
CNV	Copy-number variation
BRCA1/2	Breast Cancer gene 1/2
cfDNA	Cell-free DNA
ctDNA	Circulating tumor DNA
DNA	Deoxyribonucleic acid
EMA	European Medicines Agency
ESMO	European Society for Medical Oncology
ESR1	Estrogen Receptor 1
ET	Endocrine treatment
HER2	Human Epidermal growth factor Receptor 2
HR	Hormone Receptor
Indel	Insertion/deletion
IQR	Interquartile range
LFU	Lost to follow-up
mBC	Metastatic breast cancer
MSI	Microsatellite instability
NGS	Next-generation sequencing
NR	Not reached
OS	Overall survival
PALB2	Partner And Localizer of BRCA2
PARP	Poly (ADP-ribose) polymerase
PCR	Polymerase Chain Reaction
PIK3CA	Phosphatidylinositol-4,5-bisphosphate 3-kinase catalytic subunit alpha
PI3K	Phosphoinositide 3-kinase
PFS	Progression-free survival
PTEN	Phosphatase and TENsin homolog deleted on chromosome 10
Q1–3	First to third quartile
SERD	Selective Estrogen Receptor Degrader
SNP	Single-nucleotide polymorphism
SNV	Single-nucleotide variant
TMB	Tumor mutational burden
VAF	Variant Allele Frequency
1L	First line
