# Supplementary material for: Real-World Implementation of Molecular Testing and Biomarker-Guided Therapies in HR+/HER2− Metastatic Breast Cancer
Source: Cancers (Basel). 2026 Oct 28;18(21):3482. doi: 10.3390/cancers18213482 (PMC13761559; doi:10.3390/cancers18213482)
Supplement: Supplementary file 1 [file cancers-18-03482-s001.zip › cancers-4471675-supplementary.pdf]

Supplementary Materials

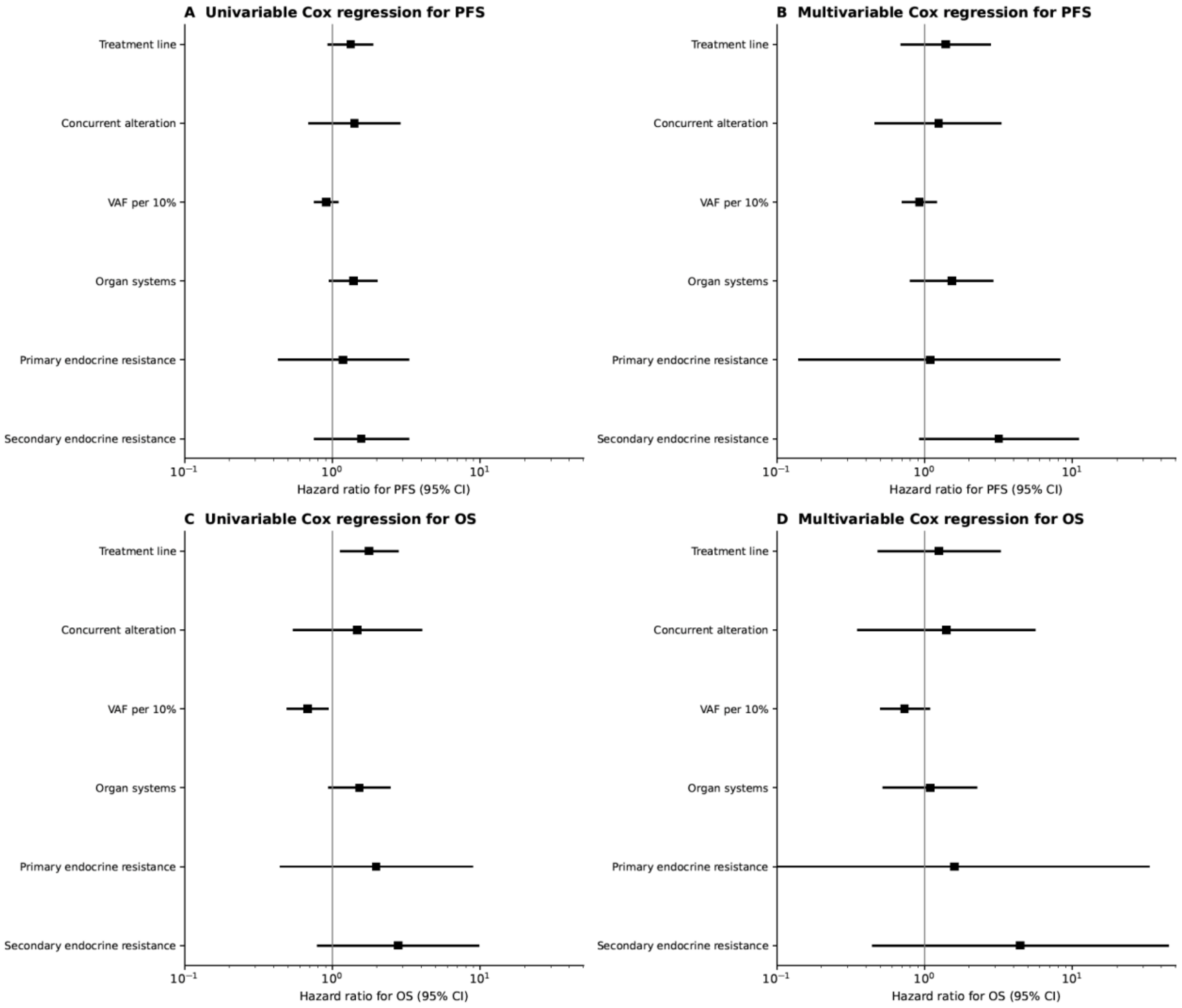

Figure S1. Forest plots of univariable and multivariable Cox regression models for progression-free and overall survival. Multivariable analyses included 34 patients with complete data for all covariates, with 26 PFS events and 15 OS events. PFS progression free survival, OS overall survival, VAF variant allele frequency.

Table S1. Overview of molecular assays used for biomarker assessment, SNV single-nucleotide variant, indel insertion/deletion, CNV copy-number variation, TMB tumor mutational burden, MSI microsatellite instability, cfDNA cell-free DNA, SNP single-nucleotide polymorphism.

Molecular assay	Specimen type	Covered alterations	PIK3CA	AKT1	PTEN	ESR1	BRCA1/2
Oncomine Comprehensive Assay v3 (Thermo Fisher Scientific)	Tissue	SNVs, indels, CNVs, gene fusions	Yes	Yes	Yes	Yes	Yes
Oncomine Focus Assay (Thermo Fisher Scientific)	Tissue	SNVs, indels, CNVs, gene fusions	Yes	Yes	No	Yes	No
TruSight Oncology 500 (Illumina)	Tissue	SNVs, indels, CNVs, gene fusions/splice variants, TMB, MSI	Yes	Yes	Yes	Yes	Yes
AmoyDx Comprehensive Panel (Amoy Diagnostics)	Tissue, blood (cfDNA)	SNVs, indels, CNVs, gene fusions, SNPs, MSI	Yes	Yes	Yes	Yes	Yes

Hedera Profiling 3 ctDNA test panel (Hedera Dx)	Blood (cfDNA)	SNVs, indels, CNVs, gene fusions, MSI	Yes	Yes	Yes	Yes	Yes
Hereditary Cancer Solution (SOPHiA Genetics)	Blood (germline DNA)	SNVs, indels, CNVs	No	No	Yes	No	Yes
Targeted PCR followed by Sanger sequencing	Tissue	Targeted sequence variants	Yes	No	No	No	No

Table S2. Characteristics of patients with and without mutation analysis, * including patients with > 1st treatment line, ET endocrine treatment, ADC antibody drug conjugate.

Patient, disease and treatment characteristics	Patients without mutation analysis, n = 100 (%)	Patients with mutation analysis, n = 174 (%)	p-value
**Age,** years, median (Q1-Q3)	67 (56-79)	59 (50-72)	<0.001
**Female sex**	100 (100%)	174 (100%)	
**Timing of metastatic disease**			0.585
De novo	36 (36.0%)	57 (32.8%)	
Recurrent	64 (64.0%)	117 (67.2%)	
**Endocrine sensitivity***			0.053
Endocrine sensitive disease	11 (57.9 %)	34 (29.6%)	
Primary endocrine resistance	2 (10.5 %)	19 (16.5%)	
Secondary endocrine resistance	6 (31.6%)	62 (53.9%)	
**Treatment line**			<0.001
1st treatment line	81 (81.0%)	59 (33.9%)	
> 1st treatment line	19 (19.0%)	115 (66.1%)	
**Types of previously received treatments**			
**1st treatment line**			
CDK4/6-inhibitor + ET	69	55	
Fulvestrant monotherapy	5	0	
Aromatase inhibitor or tamoxifen monotherapy	6	1	
**> 1st treatment line**			
CDK4/6-inhibitor + ET	17	111	
Everolimus + ET	3	51	
Fulvestrant monotherapy	6	18	
Aromatase inhibitor or tamoxifen monotherapy	13	25	
Chemotherapy	3	41	
ADC	0	17	

Table S3. Mutation analyses, continued from Table 2. Treatment line and specimen type are reported per molecular analysis.

Molecular testing	PIK3CA	PTEN	AKT1	ESR1	BRCA1/2
**Treatment line at testing,** median (Q1-Q3)	1 (0, 2)	1 (0, 2)	1 (0, 3)	2 (1, 3)	NA
**Specimen type**					
Blood	11	1	2	13	14
Bone	21	0	1	5	0
Liver	8	3	0	1	0

Lymph nodes	6	0	0	1	0
Lung	2	0	0	0	0
Other visceral sites	34	6	2	7	0
missing	2	0	0	0	0

Table S4. Utilization and treatment characteristics of biomarker-guided therapies. Survival analyses were performed according to the first biomarker-guided therapy received, ET endocrine treatment.

Therapy	Patients, n	Treatment line, median (Q1-Q3)	PFS, months (95% CI)	OS, months (95% CI)
**Alpelisib + ET**	29	2 (2-2)	10.9 (7.5–14.0)	47.8 (12.3–NR)
**Capivasertib + ET**	4	3 (2-5)	5.2 (3.4–NR)	NR
**Elacestrant**	16	3 (2-3)	3.5 (0.6–5.1)	9.7 (0.6–NR)
**PARP inhibitor**	8	2 (2-2)	14.5 (3.4–NR)	34.3 (3.4–NR)
**No biomarker-matched therapy**	47	-	-	-
